# Dendrimer-modified carbon nanotubes for the removal and recovery of heavy metal ions from water

**DOI:** 10.3762/bjnano.16.107

**Published:** 2025-09-01

**Authors:** Thao Quynh Ngan Tran, Huu Trung Nguyen, Subodh Kumar, Xuan Thang Cao

**Affiliations:** 1 Faculty of Chemical Engineering, Industrial University of Ho Chi Minh City, Vietnam; 2 Department of Inorganic Chemistry, Faculty of Science, Palacký University, 17 Listopadu 12, CZ-77146 Olomouc, Czech Republic

**Keywords:** carbon nanotubes, deep eutectic solvent, dendrimers, Diels–Alder reaction, heavy metal ion adsorption

## Abstract

Effective removal of trace heavy metal ions from aqueous bodies is a pressing problem and requires significant improvement in the area of absorbent material in terms of removal efficiency and sustainability. We propose an efficient strategy to enhance the adsorption efficiency of carbon nanotubes (CNTs) by growing dendrimers on their surface. First, CNTs were pre-functionalized with maleic acid (MA) via Diels–Alder reaction in presence of a deep eutectic solvent under ultrasonication. Subsequently, dendrimers of varying length were grown by the repeated reaction of ethylene diamine and MA. Raman spectroscopy was specifically used to confirm the Diels–Alder reaction on the surface of CNTs, and other characterization techniques (SEM, EDX, XRD, TGA, and FTIR) were applied to confirm the successive growth of the dendrimers. Highly dendrimerized CNTs were found to be more effective in removing heavy metal ions (Pb^2+^ and Cd^2+^) from aqueous solutions with enhanced recyclability than less dendrimerized CNTs. Kinetic studies have revealed that the adsorption process followed a pseudo-second order kinetic model, and the rate-limiting step was mainly chemisorption. This study has not only excluded the involvement of harmful chemicals to pre-functionalize the CNTs with high loading but also provided an effective way to enhance the adsorption of heavy metal ions.

## Introduction

The contamination of water bodies with heavy metal ions poses a significant threat to the environment and human health due to their non-degradable nature and toxicity. Metal-oriented industries such as battery manufacturing, mining, electroplating, and metal recycling are growing day by day, and so are the chances of metal contamination. Consequently, the efficient removal of heavy metal ions remains a critical research challenge. Various methods have been employed including membrane filtration [1], flocculation [2], adsorption [3–4], precipitation [5], electrolytic removal [6], ion exchange [7], reduction [8], and reverse osmosis [9]. The adsorption process stands out as a promising approach due to its high efficiency, cost-effectiveness, ease of processing, and capability to recover trace metal ions [10–13]. This process utilizes nanomaterials bearing appropriate functional groups to bind metal ions, which can be later released by altering the pH of the solution. Numerous nanomaterials have been explored and modified by introducing a high number of binding sites (functional groups) and/or generating a porous structure to enhance the adsorption of heavy metal ions [14–15]. Although such modified materials have shown improved performance, the modification processes are generally energy-intensive and require harmful chemicals. Moreover, the optimum incorporation of chemical functionality, responsible for binding the trace metal ions, still needs to be achieved. Therefore, it is always a race to develop more economical and sustainable adsorbent materials. Dendrimers are hyperbranched polymeric materials whose chemical functionality as well as the length of polymeric branches can be controlled. These properties make them suitable candidates for the adsorption process. Moreover, the growth of such dendrimers on a solid support can also improve the dispersibility, accessibility of binding sites, chemical recyclability, and mechanical stability [16–17]. In fact, dendrimers have been supported by various nanomaterials such as silica, graphene oxide, and carbon nanotubes (CNTs), broadening their application in numerous fields [18–23]. CNTs are particularly attractive as support materials due to their high specific surface area, mechanical strength, and excellent electrical and thermal conductivity [24–27]. CNTs themselves have been utilized as adsorbents for removing heavy metals from water solution [28–30]. However, the poor dispersibility of bare CNTs in most solvents including water is a significant limitation to their wider applicability. Covalent modification of CNT surfaces with dendrimers of desirable functionality can improve their dispersibility and binding efficiency. Generally, dendrimer growth on CNT surfaces requires pre-functionalization through radiation or chemical oxidation processes involving strong acids and oxidants [31–35]. However, these processes can damage CNTs, thus diminishing their core properties and generating large amounts of acidic waste at the same time. Covalent functionalization of dendrimers on CNT surfaces via Diels–Alder reactions transforming sp^2^ carbon into sp^3^ carbon is a desirable alternative. This technique has been successfully used to generate various functionalities on CNT surfaces using different organic moieties and polymers; however, it typically involves harmful organic solvents (e.g., *N*-methyl-2-pyrrolidone and dimethylformamide) and reagents [36–38]. Fortunately, deep eutectic solvents (DESs) have been identified as suitable catalytic media for Diels–Alder reactions without the need for external reagents or solvent systems [39–40]. DESs are formed through hydrogen bonding between two solid components resulting in eutectic liquids having a melting point lower than those of the individual components [41–42]. They offer advantages such as simple synthesis, low cost, and broad applications in biosynthesis, extraction processes, and organic reactions [43].

In this study, we focus on the pre-functionalization of CNTs via Diels–Alder reaction using DES as the sole catalytic system under sonication energy and subsequent growth of dendrimers. We further evaluate the potential of the CNTs in the removal of trace heavy metals from aqueous solutions explaining the kinetics and thermodynamics of adsorption process.

## Results and Discussion

**Scheme 1:**
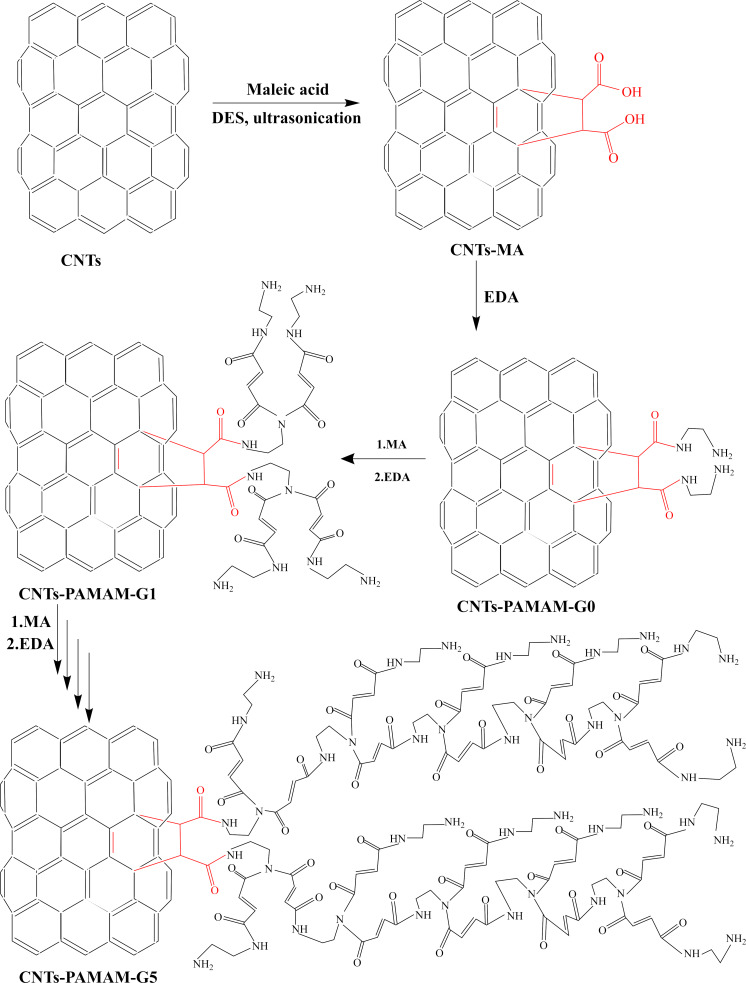
Preparation of CNTs-PAMAM dendrimer.

**Figure 1:**
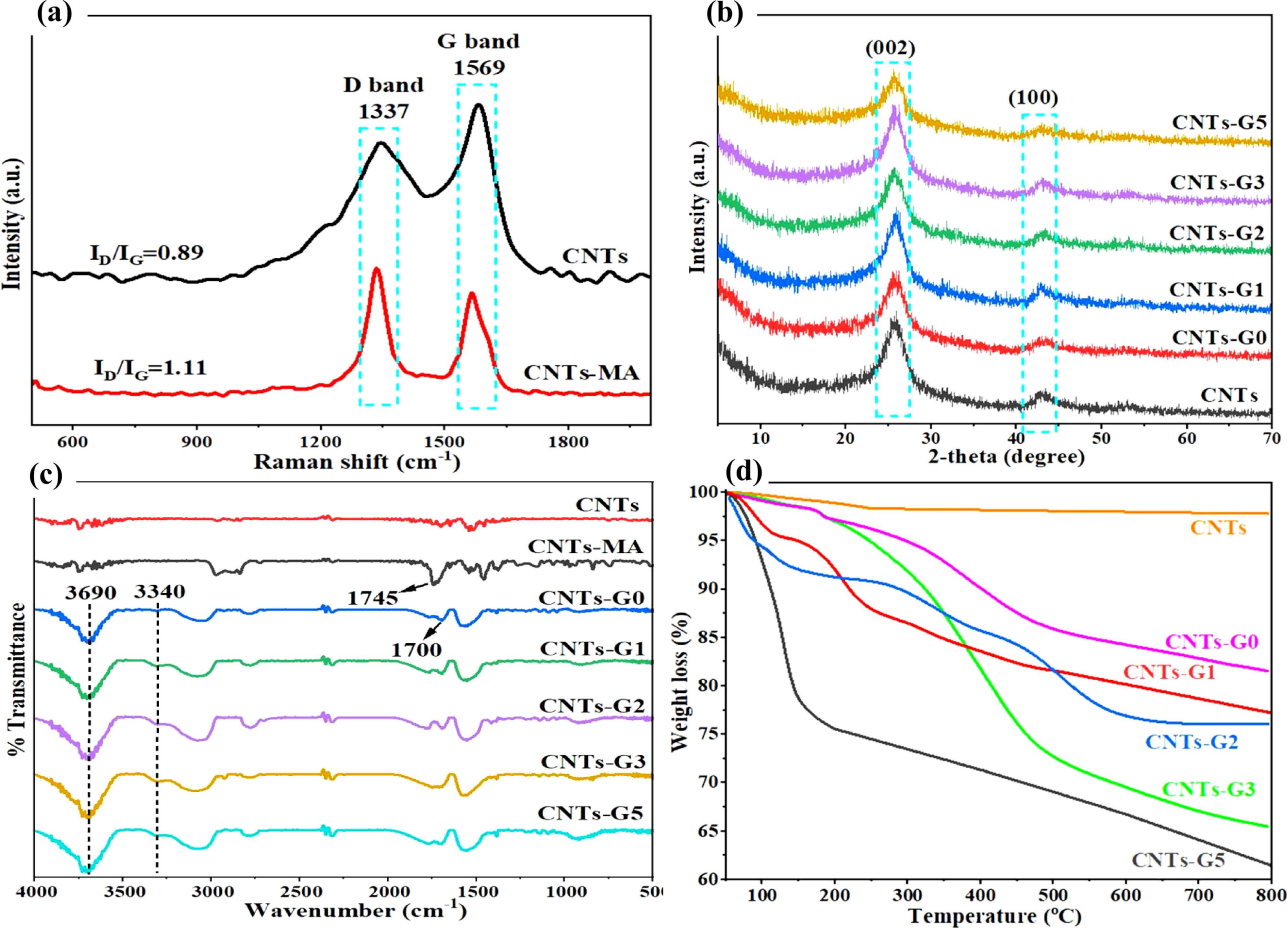
(a) Raman spectra of CNTs and CNTs-MA; (b) diffractograms of CNTs, CNTs-G0, CNTs-G1, CNTs-G2, CNTs-G3, and CNTs-G5; (c) FTIR spectra of CNTs, CNTs-MA, CNTs-G0, CNTs-G1, CNTs-G2, CNTs-G3, and CNTs-G5; and (d) TGA curves of CNTs, CNTs-G0, CNTs-G1, CNTs-G2, CNTs-G3, and CNTs-G5.

Dendrimerized CNTs have been fabricated by pre-functionalizing the CNTs with MA via Diels–Alder reaction, excluding harmful chemicals and energy-intensive processes (Scheme 1). This first step is crucial to ensure a high growth rate of dendrimers in subsequent steps by the repeated reaction of MA and ethylene diamine (EDA). We have characterized the CNTs-MA material by Raman spectroscopy to find the degree of functionalization, and the results are depicted in Figure 1a. The Raman spectrum exhibits two characteristic peaks, namely, the D peak at 1337 cm^−1^ and the G peak at 1569 cm^−1^. The ratio of the intensities of D and G peaks (*I*_D_/*I*_G_) has increased from 0.89 to 1.11 after MA functionalization, indicating a successful transformation of carbon atoms from sp^2^ to sp^3^ on the surface of CNTs. Accordingly, it reflects the reduction of double bonds on the CNT surfaces, thus indicating the formation of new covalent bonds between MA and CNTs. The further growth of dendrimers was achieved by the successive reaction of EDA and MA under the reaction conditions described in the Experimental section. Finally, all materials were characterized by various other techniques which are discussed thoroughly in the following sections. XRD analysis of CNTs, CNTs-G0, CNTs-G1, CNTs-G2, CNTs-G3, and CNTs-G5 presented two prominent peaks at 2θ ≈ 26° and 43° (Figure 1b). The first peak, with higher intensity, corresponds to the (002) plane of graphite indicating a d-spacing (*d*_002_) of 0.34 nm, and the second peak ° is associated with the (100) planes of graphite. Both peaks are characteristic to the graphitic crystal lattice of CNTs [44]. Hence, XRD results indicate that the functionalization process did not alter the intrinsic structure of the materials as the peak profile of all the samples is similar to that of bare CNTs. Moreover, the absence of any extra peaks further confirms that the washing process has effectively removed the residual substrates and DES.

FTIR spectroscopy has provided additional insights into the functionalization process (Figure 1c). The FTIR spectrum of CNTs-MA shows a peak around 1745 cm^−1^ corresponding to the C=O stretching of the carboxyl groups due to the introduction of MA, thus indicating successful covalent grafting onto the surface of CNTs. After reaction with EDA, the FTIR spectrum of CNTs-G0 reveals new peaks around 3340 cm^−1^, which correspond to N–H stretching vibrations from the introduced amine groups. Additionally, strong absorption bands at approximately 1700 cm^−1^ were observed in all other materials, representing the C=O stretching of amide bonds. Moreover, the presence of a band at 3690 cm^−1^, associated with the stretching of amine groups overlapped with hydroxy stretching vibrations, further confirms the successful formation of amide linkages by the reaction between amine groups of EDA and carboxyl groups of MA-functionalized CNTs [45]. These results collectively indicate that the functionalization process has effectively introduced –NH_2_ and amide groups onto the CNT surfaces.

TGA was utilized to further confirm the polymeric growth of dendrimers on the surface of CNTs (Figure 1d). TGA results indicated a minor weight loss between 100 and 150 °C for the unfunctionalized CNTs, which is attributed to the release of physically adsorbed water. Moreover, bare CNTs exhibited thermal stability up to 800 °C with a residual weight of 97.80% suggesting the absence of organic functional groups. In contrast, CNTs functionalized with MA and EDA (CNTs-G0) exhibited a distinct thermal behavior displaying an initial weight loss around 150 °C, which is likely due to the loss of water, followed by significant thermal degradation around 500 °C, corresponding to the decomposition of carboxyl groups.

The CNTs-G0 sample retained 81.58% of its weight at 800 °C, indicating significant thermal stability, but surely less than unfunctionalized CNTs due to the presence of organic functional groups. Further thermal analysis of CNTs functionalized with dendrimers such as CNTs-G1, CNTs-G2, CNTs-G3, and CNTs-G5 have shown more pronounced weight losses at different temperature ranges. Weight loss due to the physically adsorbed water can be observed around 150 °C. However, significant weight loss occurred between 150 and 600 °C due to the decomposition of carboxylic acid, amide groups, and anhydrides as well as dehydration processes. The further weight losses above 500 °C could be due to the breakdown of carbon bonds to reconstruct the more stable graphitic structures. The residual weight percentages of CNTs-G1, CNTs-G2, CNTs-G3, and CNTs-G5 at 800 °C were 77.28%, 76.09%, 65.49%, and 61.57%, respectively, indicating a successive decrease in thermal stability with increasing dendrimer functionalization.

**Figure 2:**
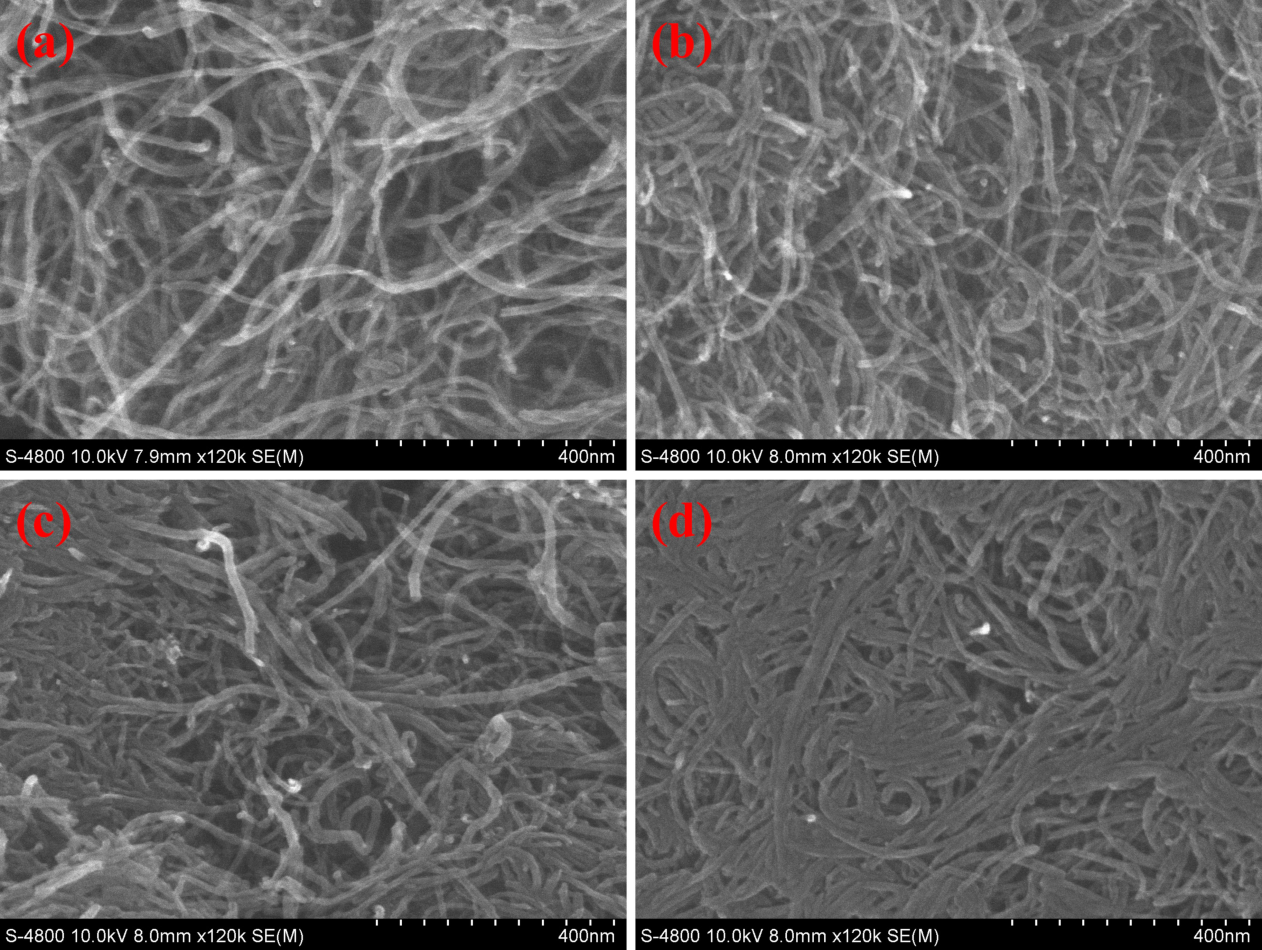
SEM images of (a) CNTs, (b) CNTs-G1, (c) CNTs-G3, and (d) CNTs-G5.

SEM images are helpful to identify the morphological changes after the functionalization of CNTs. The comparative observation of SEM images of CNTs, CNTs-G1, CNTs-G3, and CNTs-G5 clearly reveals tube shape structure for all the materials confirming no morphological change during the dendrimer growth process. Moreover, there were no carbon particulates associated with residual carbon polymerization indicating uniform dendrimer growth over the surface of CNTs. However, changes in terms of degree of aggregation were observed (Figure 2). The aggregation among the CNTs was found to be increased with the successive growth of dendrimers resulting in dense carbon nanotubes. It could be due to the interactions such as hydrogen bonding between the growing number of functional groups on the surface of CNTs because of successive growth of dendrimers. Moreover, elemental composition of the bare CNTs and their functionalized derivatives have been revealed by the EDX spectroscopic analysis (Supporting Information File 1, Figure S1). Pristine CNTs were found to have 97.79% carbon and 2.21% oxygen reflecting the primarily carbon-based structure with minimal oxygen content. It is evident that there is a successive increase in the atomic percentage of C– and N– in the respective materials. CNTs-G1, CNTs-G3 and CNTs-G5 samples comprised 70.01% of C and 5.37% of N, 80.81% of C and 8.31% of N, and 82.25% of C and 12.29% of N respectively. This observed trend signifies the successful successive growth of branched polymeric dendrimers across the respective samples. Moreover, the presence of N– element clearly indicates the involvement of EDA in subsequent polymerization reactions.

**Figure 3:**
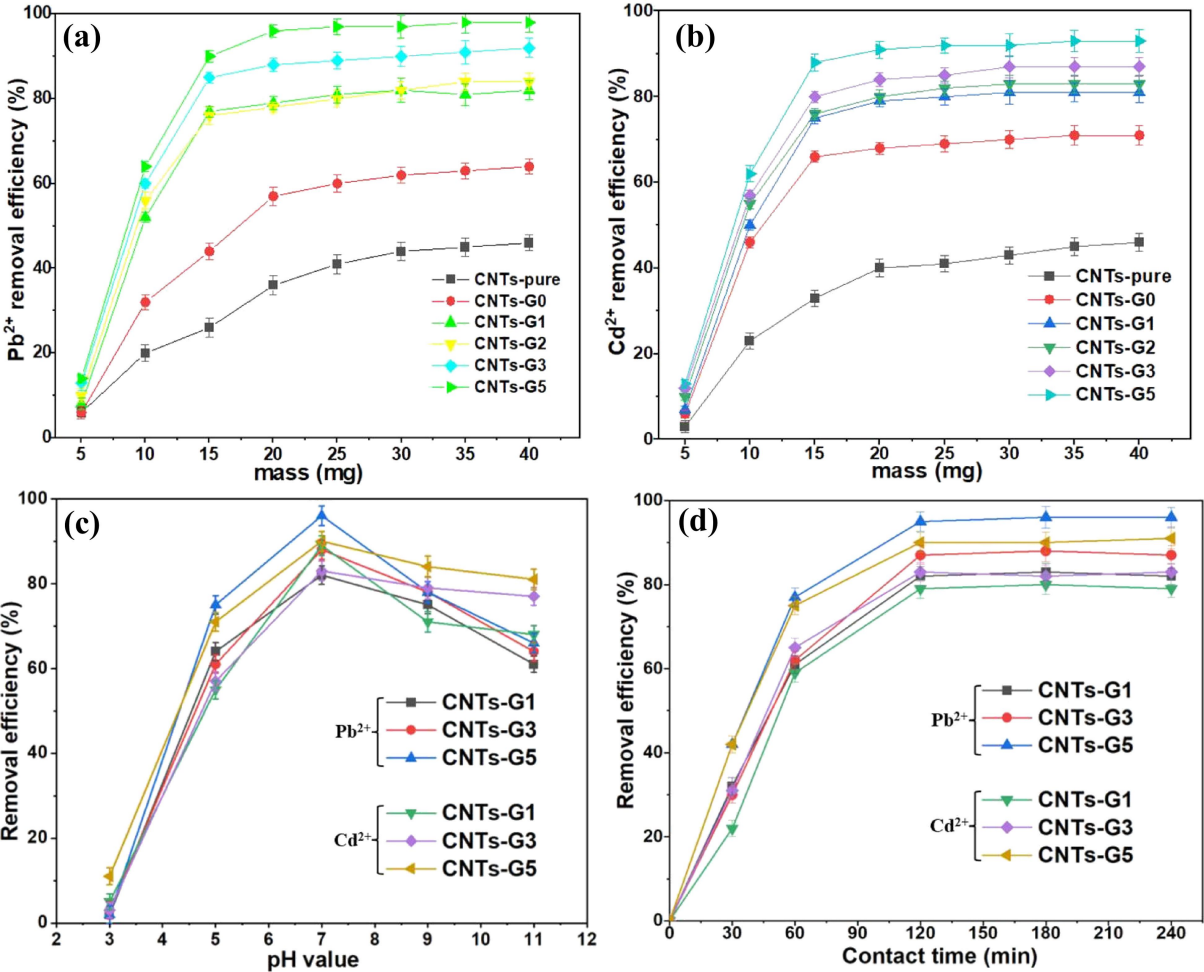
Effect of (a, b) adsorbents dosage, (c) solution pH, and (d) contact time on the removal efficiency of dendrimer-functionalized CNTs towards Pb^2+^ and Cd^2+^ ions. Each data point represents the average of three independent experiments; error bars indicate standard deviations (*n* = 3).

A trace metal adsorption study was conducted using the dendrimerized CNTs as described in the Experimental section. First, 10 mg of the synthesized dendrimerized CNTs (CNTs-G1, CNTs-G2, CNTs-G3, and CNTs-G5) with different degrees of branching were added to respective vials as adsorbents, each containing 20 mL of a 100 ppm solution of Pb^2+^ and Cd^2+^ diluted from a 1000 ppm stock. The resulting dispersions were sonicated followed by stirring at room temperature for 2 h to facilitate the adsorption process in neutral medium. A successive increase in adsorption efficiency of dendrimerized CNTs from CNTs-G1 to CNTs-G5 was observed, clearly indicating the significant role of stepwise growth of highly branched dendrimers. CNTs-G5 as adsorbent provide a high number of binding sites in the form of functional groups (–NH_2_ and –CO–NH–) and have a high surface area, allowing facile accessibility to more metal ions. Moreover, we have investigated the effect of various parameters such as the added mass of the dendrimerized CNTs (adsorbent), pH of the aqueous medium, and reaction time of the adsorption process, and the results are summarized in Figure 3. The adsorption efficiency of the dendrimerized derivatives CNTs-G1 to CNTs-G5 improved from as their mass was increased from 5.0 to 20.0 mg (Figure 3a,b). However, the adsorption efficiency plateaued when the mass was further increased from 20.0 to 40.0 mg. Based on these findings, the optimal mass of adsorbent was selected to be 20.0 mg for the subsequent adsorption experiments. Further, the influence of pH of the aqueous medium on the adsorption efficiency of adsorbents was studied (Figure 3c). The adsorption efficiency for both ions was found to be increased as the pH was raised from 3.0 and reached a maximum at pH 7.0. This trend can be explained by the fact that the high concentration of H^+^ ions at lower pH values might be competing with Pb^2+^ and Cd^2+^ ions for adsorption sites. In contrast, higher pH values above 7.0 caused the adsorption efficiency to decrease, which could be due to the formation of metal hydroxide precipitates. Consequently, pH 7.0 was determined to maximize the adsorption efficiency of dendrimerized CNT adsorbents for both Pb^2+^ and Cd^2+^ ions.

The impact of contact time on the Pb^2+^ and Cd^2+^ ion adsorption efficiency was also investigated (Figure 3d). Each adsorbent showed increased adsorption efficiency for both the metal ions in the case of longer contact time ranging from 0 to 240 min. This behavior suggests that longer time is favorable for metal ions to be adsorbed onto the surface of dendrimerized CNTs. However, the adsorption efficiency reached a plateau after 120 min, implying an equilibrium between adsorption and desorption processes. Accordingly, 120 min was chosen as the optimal contact time for further adsorption studies.

**Figure 4:**
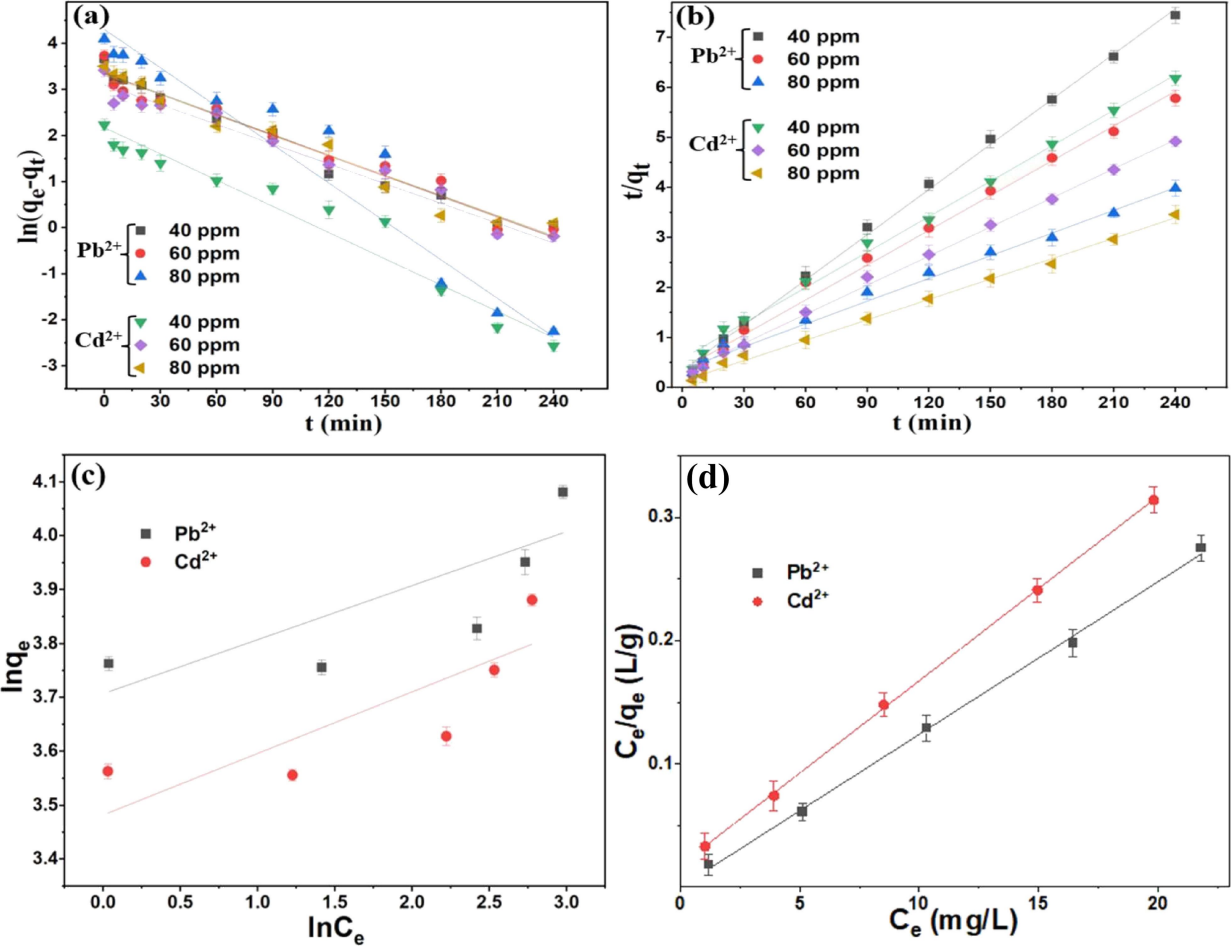
Kinetics and isotherm models for metal ion adsorption onto the CNTs-G5: (a) Pseudo-first order, (b) pseudo-second order, (c) Freundlich isotherm, and (d) Langmuir isotherm models.

The removal of metal ions (Pb^2+^ and Cd^2+^) by dendrimer-functionalized CNTs is primarily governed by physical interactions. However, the nitrogen-rich surface of the poly(amidoamine) (PAMAM) dendrimers offers multiple coordination sites, which might facilitate the chelation of divalent ions at later stages. In fact, chelation mechanism has been reported in previous studies including PAMAM-functionalized carbon materials [46–48]. Moreover, the kinetic study for the adsorption of Pb^2+^ and Cd^2+^ metal ions by dendrimerized CNTs-G5 was evaluated using two kinetic models: pseudo-first order and pseudo-second order. These models have been described by their respective equations (Supporting Information File 1, Equations S1 and S2). For the pseudo-first order kinetic model, a linear plot of ln(*q*_e_ − *q*_t_) as a function of time (*t*) was drawn at different initial concentrations of metal ions (Figure 4a and Supporting Information File 1, Table S1). The correlation coefficients were calculated to be relatively low. In contrast, the pseudo-second order kinetic model represented by the plot of *t*/*q*_t_ against time (*t*) demonstrated a much better fit with higher correlation coefficients across all concentration levels (Figure 4b and Supporting Information File 1, Table S1). Moreover, average relative error (ARE) values for the pseudo-first order model were higher than those for the pseudo-second order model. The pseudo-second order model implies that the metal ion adsorption is controlled by chemical interactions rather than physical interactions alone [49–50]. Although the pseudo-second order model is often associated with surface-controlled or chemical interactions, its application does not necessarily confirm chemisorption. In this study, thermodynamic analysis (Supporting Information File 1, Table S4) showed relatively low enthalpy changes (Δ*H* < 80 kJ/mol), which are more consistent with physisorption rather than chemisorption (typically 80–400 kJ/mol). Thus, the adsorption of Pb^2+^ and Cd^2+^ onto CNTs-G5 is better interpreted as being dominated by physisorption, potentially accompanied by weak electrostatic or coordination interactions with dendrimer surface groups.

Isotherm studies can provide important information about the metal ion adsorption process. Therefore, Freundlich and Langmuir isotherm models have been applied (Supporting Information File 1, Equations S4 and S5). Low correlation coefficient values (*R*^2^ = 0.6985 for Pb^2+^ and *R*^2^ = 0.7066 for Cd^2+^) were obtained for the Freundlich model, which indicate poor fit with the experimental data of the metal ion adsorption on CNTs-G5. In contrast, the experimental data was fitted better with high correlation coefficients (*R*^2^ = 0.9984 for Pb^2+^ and *R*^2^ = 0.9997 for Cd^2+^) when the Langmuir isotherm model was applied. This implies monolayer adsorption onto a homogeneous surface. Pb^2+^ and Cd^2+^ ions might have adsorbed onto the CNTs-G5 surface in a uniform layer without further adsorption once the surface gets saturated. Moreover, the applicability of Langmuir model confirms that the adsorption occurs on specific, identical sites of the CNTs-G5 surface (Figure 4c,d and Supporting Information File 1, Table S2). A recent study has observed multilayer adsorption in graphene/CNT-PAMAM hybrid materials [48], supported by Freundlich isotherm fitting and heterogeneous surface characteristics. However, the adsorption behavior can vary on many factors such as type of adsorbates, adsorbent composition, reaction conditions, and degree of functionality. In our case, the adsorption behavior of pure CNT-based dendrimer adsorbents appears to be predominantly monolayer in nature. Importantly, the maximum adsorption capacities (*q*_max_) derived from the Langmuir model, 80.71 mg/g for Pb^2+^ and 66.80 mg/g for Cd^2+^, were found to be higher than those of other reported adsorbents for these metal ions (Supporting Information File 1, Table S3). Furthermore, thermodynamic parameters such as changes of enthalpy (Δ*H*), entropy (Δ*S*), and Gibbs free energy (Δ*G*) were calculated by plotting the graph of ln *K*_e_ vs 1/*T* and using the van't Hoff equation (Supporting Information File 1, Figure S2 and Table S4). Positive Δ*H* and negative Δ*G* values confirm that metal ion adsorption is an endothermic spontaneous process across all studied temperatures. The relatively low Δ*H* values support the conclusion that the overall adsorption mechanism is primarily governed by physisorption. The recyclability of the adsorbents is an important parameter to assess their suitability for the long-term application to reduce the cost of the adsorption process. The recycling performance of CNTs-G5 has been presented in the form of removal efficiency in five consecutive cycles (Supporting Information File 1, Figure S3). It is evident from the results that CNTs-G5 performed well as an effective adsorbent securing adsorption efficiencies of 81.9 and 74.3% for Pb^2+^ and Cd^2+^ metal ions, respectively. Adsorbent materials were treated with HNO_3_ to desorb the metal ions and washed repeatedly with deionized water until neutral pH and then dried after every cycle before use.

## Conclusion

In this study, we successfully functionalized MA onto the surface of CNTs via Diels–Alder reaction, utilizing a DES as a catalytic medium. This functionalization provided a facile platform for further successive reactions of EDA and MA to grow multibranched polymer networks. Dendrimerized CNTs were thoroughly characterized using FTIR, SEM, XRD, EDX, TGA, and Raman spectroscopy to confirm the successful functionalization and structural integrity of the materials. The applicability of these dendrimerized CNTs materials for the adsorption of Pb^2+^ and Cd^2+^ metal ions from aqueous media was thoroughly investigated. The highest adsorption efficiencies for both Pb^2+^ and Cd^2+^ metal ions were achieved with CNTs-G5 under optimal conditions (pH 7.0, adsorption time 2 h, and 20 mg weight). Moreover, recyclability tests have shown that CNTs-G5 can be reused for up to five cycles while maintaining adsorption efficiencies of 81.9 and 74.3% for Pb^2+^ and Cd^2+^ metal ions, respectively, affirming its potential use for practical applications. Based on kinetic studies, the adsorption process was best described by a pseudo-second order reaction, and the rate-limiting step was mainly chemisorption. Calculated value of thermodynamic parameters such as Δ*H* and Δ*G* asserted that adsorption process was endothermic and spontaneous in nature.

## Experimental

### Materials

Multiwalled carbon nanotubes (CNTs) (CM-95, diameter 10–15 nm, length 10–20 μm, specific surface area 200 m^2^/g) were purchased from Hanwha Nanotech, Korea. Ethylenediamine and maleic anhydride (99%) were obtained from Alfa Aesar and used as received without further purification. Standard solutions of Pb and Cd (1000 ppm) were procured from Merck with a purity of 99.99% and used as received.

### Characterizations

Raman spectroscopy was conducted using a JASCO NRS-5000 spectrometer with a 532 nm diode laser to detect structural changes and defects in the CNTs after Diels–Alder reaction. FTIR spectroscopy was employed using a Bruker Tensor 27 spectrometer in the 4000–500 cm^−1^ region, which confirmed the change in functional groups during dendrimer functionalization. XRD patterns were recorded on a Shimadzu 6100 X-ray diffractometer in a 2θ range of 10–80°, providing insights into the crystalline structure and phase composition of the CNTs before and after modification. UV–vis spectroscopy was used to monitor adsorption of metal ions by the interaction of dendrimerized CNTs. Additionally, atomic absorption spectroscopy was employed to determine the concentration of heavy metal ions in the aqueous solutions before and after the adsorption process.

### Preparation of dendrimerized carbon nanotubes

20 g of maleic anhydride was first hydrolyzed into maleic acid at 70 °C using 30 mL of distilled water through a ring-opening mechanism [51]. Then, 0.4 g of freshly prepared MA was mixed with 3.0 g of a DES synthesized by the reaction of choline chloride and zinc chloride in a 1:2 molar ratio under continuous stirring at room temperature until a homogeneous liquid was formed [52]. The mixture of MA and DES was stirred at room temperature for 10 min and then diluted with 3 mL of deionized water to obtain a homogeneous solution. Subsequently, 100 mg of pure CNTs were gradually added to the MA-DES solution under continuous magnetic stirring for 30 min to ensure proper wetting and dispersion. The resulting suspension then underwent ultrasonication at 60 °C for 4 h using a bath sonicator (40 kHz, 150 W) to facilitate the Diels–Alder reaction between the CNT surface and MA. The resulting CNTs functionalized with MA (CNTs-MA) were collected by vacuum filtration using a 0.45 µm PTFE membrane, then thoroughly washed several times with deionized water followed by ethanol to remove any unreacted MA and residual DES. The washed material was subsequently dried in an oven at 45 °C overnight. The CNTs-MA (100 mg) was dispersed in 10 mL of methanol under a nitrogen atmosphere and sonicated for 10 min to ensure uniform suspension. Then, 0.2 mL of deoxygenated EDA was slowly injected under continuous stirring. The mixture was stirred at room temperature for 24 h under inert conditions. After completion, the resulting solid material was collected by filtration, washed thoroughly with methanol and deionized water, and dried at 45 °C to obtain the first-generation dendrimer-functionalized CNTs, and labelled as CNTs-G0. This material served as the base for subsequent dendrimer growth through alternating reactions with MA and EDA. To initiate dendrimer growth, CNTs-G0 (100 mg) and MA (0.3 g) were dispersed in 3.0 mL of methanol at room temperature and stirred overnight to allow carboxylation. The product was collected by vacuum filtration, washed thoroughly with ethanol, and dried at 45 °C to obtain the second-generation dendrimer-functionalized sample, denoted as CNTs-G1. Subsequent generations (CNTs-G2, CNTs-G3, and CNTs-G5) were synthesized by repeating alternating reactions with 0.3 g of MA and 0.2 mL of EDA under the same conditions as described for G1. The detailed step-by-step procedure is indicated in Scheme 1. The isolated yields for each synthetic step were approximately 85% (CNTs-MA), 81% (CNTs-G0), 78% (CNTs-G1), 73% (CNTs-G2), 68% (CNTs-G3), and 59% (CNTs-G5).

### Adsorption of heavy metal ions

Dendrimer-functionalized CNTs were tested to adsorb heavy metal ions from aqueous solutions. Specifically, the required amounts of the synthesized materials were added to separate vials, each containing 20 mL of a 100 ppm working solution of Pb^2+^ and Cd^2+^ ions, prepared by diluting a 1000 ppm standard stock solution with deionized water. The mixtures were first sonicated for 10 min to ensure uniform dispersion and effective interaction between the adsorbents and the metal ions. Subsequently, the dispersions were magnetically stirred for another 2 h at room temperature to facilitate the adsorption process. Afterward, the solid adsorbents were separated by centrifugation, and re-dispersed in 10 mL of 1 M HNO_3_ solution to desorb the metal ions. The mixture was stirred for an additional 2 h at room temperature. The remaining concentrations of Pb^2+^ and Cd^2+^ in solution after the adsorption process were determined using atomic absorption spectroscopy. Finally, the recovered dendrimerized CNTs were thoroughly washed with deionized water until neutral pH, thus making them ready for subsequent recycling experiments. All adsorption experiments were performed in triplicate, and the results are presented as mean values with standard deviations.

## Supporting Information

File 1 Kinetic and adsorption model equations, comparison of various adsorbents for Pb^2+^ and Cd^2+^ adsorption with previously reported literature.

## Data Availability

All data that supports the findings of this study is available in the published article and/or the supporting information of this article.
