# Supplementary material for: Dendrimer-modified carbon nanotubes for the removal and recovery of heavy metal ions from water
Source: Beilstein J Nanotechnol. 2025 Sep 1;16:1522–32. doi: 10.3762/bjnano.16.107 (PMC12415912; doi:10.3762/bjnano.16.107)
Supplement: File 1 — Kinetic and adsorption model equations, comparison of various adsorbents for Pb2+ and Cd2+ adsorption with previously reported literature. [file Beilstein_J_Nanotechnol-16-1522-s001.pdf]

BEILSTEIN JOURNAL OF NANOTECHNOLOGY

Supporting Information

for

Dendrimer-modified carbon nanotubes for the removal and recovery of heavy metal ions from water

Thao Quynh Ngan Tran, Huu Trung Nguyen, Subodh Kumar and Xuan Thang Cao

Beilstein J. Nanotechnol. **2025,** *16,* 1522–1532. doi:10.3762/bjnano.16.107

Kinetic and adsorption model equations, comparison of various adsorbents for Pb^{2+} and Cd^{2+} adsorption with previously reported literature

License and Terms: This is a supporting information file under the terms of the Creative Commons Attribution License (https://creativecommons.org/licenses/by/4.0). Please note that the reuse, redistribution and reproduction in particular requires that the author(s) and source are credited and that individual graphics may be subject to special legal provisions.
The license is subject to the *Beilstein Journal of Nanotechnology* terms and conditions: (https://www.beilstein-journals.org/bjnano/terms)

Kinetic study

The pseudo-first order (Equation S1) and pseudo-second order (Equation S2) models are expressed as follows [1]:

$$\ln(q_e - q_t) = -k_1 t \quad \text{(S1)}$$

$$\frac{t}{q_t} = \frac{1}{k_2 q_{\mathrm{e}}^2} + \frac{t}{q_{\mathrm{e}}} \quad \text{(S2)}$$

where q_t and q_{e} are the amount of metal ion adsorbed per unit mass of adsorbent at time t and equilibrium (mg/g), and k_1 (1/min), and k_2 (g/mg·min) are the equilibrium rate constants. The kinetic results were also evaluated using the average relative error (ARE) as given following equation:

$$\mathrm{ARE} = \frac{1}{N}\sum_{i=1}^{N}\left|\frac{q_{\mathrm{e,exp}} - q_{\mathrm{e,cal}}}{q_{\mathrm{e,cal}}}\right| \quad \text{(S3)}$$

where $q_{\mathrm{e,exp}}$ and $q_{\mathrm{e,cal}}$ values are determined by experiment and theory, respectively.

Freundlich and Langmuir models

$$\ln q_{\mathrm{e}} = \ln K_{\mathrm{F}} + \frac{1}{n}\ln C_{\mathrm{e}} \quad \text{(S4)}$$

$$\frac{C_{\mathrm{e}}}{q_{\mathrm{e}}} = \frac{1}{K_{\mathrm{L}} q_{\mathrm{max}}} + \frac{C_{\mathrm{e}}}{q_{\mathrm{max}}} \quad \text{(S5)}$$

where, q_{e} (mg/g), C_{e} (mg/L), and q_{max} (mg/g) are the equilibrium adsorption capacity, equilibrium concentration, and maximum adsorption capacity, respectively; K_{F} (mg/g(L/mg)$^{1/n}$) and n are the Freundlich constants; K_{L} (L/mg) is the Langmuir constant.

Thermodynamic parameters

Thermodynamic parameters including changes of Gibbs free energy (ΔG), enthalpy (ΔH), and entropy (ΔS) of the adsorption can be calculated by experimental data at different temperature values following equations:

$$\Delta G = -RT\ln K_{\mathrm{e}} \quad \text{(S6)}$$

$$\ln K_e = -\frac{\Delta H}{RT} + \frac{\Delta S}{R} \quad \text{(S7)}$$

where R is the gas constant (8.314 J/mol·K), K_e is the thermodynamic equilibrium constant (L/mol), and T is the absolute temperature (K). Values of K_e may be obtained using the following equation:

$$K_e = \frac{q_e}{C_e} \quad \text{(S8)}$$

where q_e (mg/g) and C_e (mg/L) are the equilibrium concentration of metal ions on the CNTs-G5 and liquid phase, respectively.

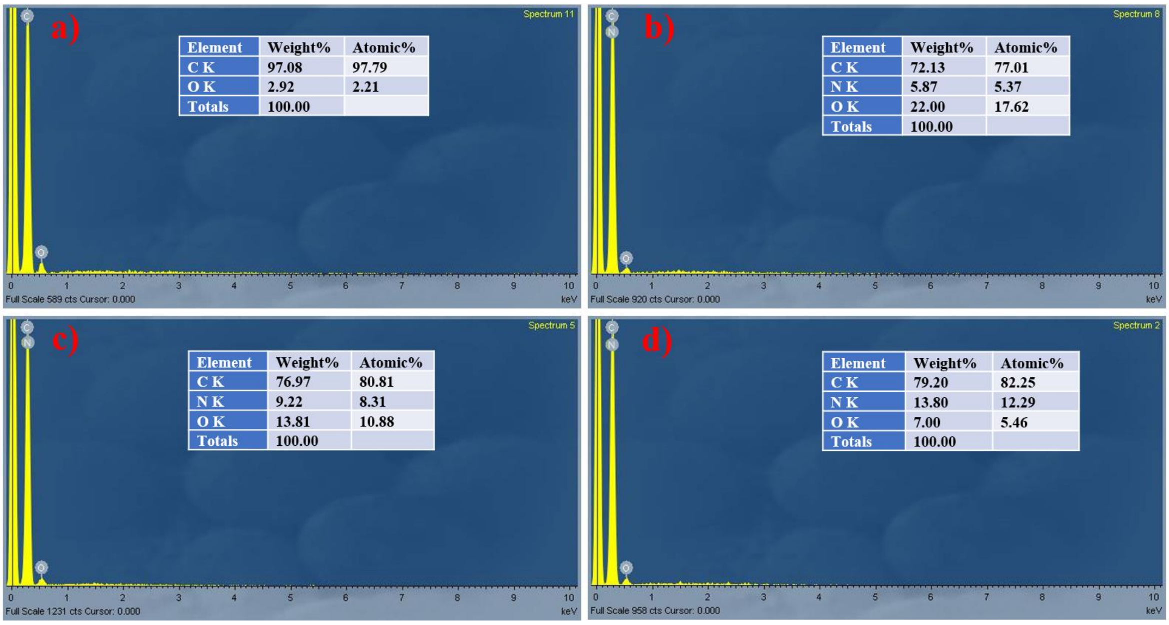

Figure S1: EDX analysis of (a) CNTs, (b) CNTs-G1, (c) CNTs-G3, and (d) CNTs-G5.

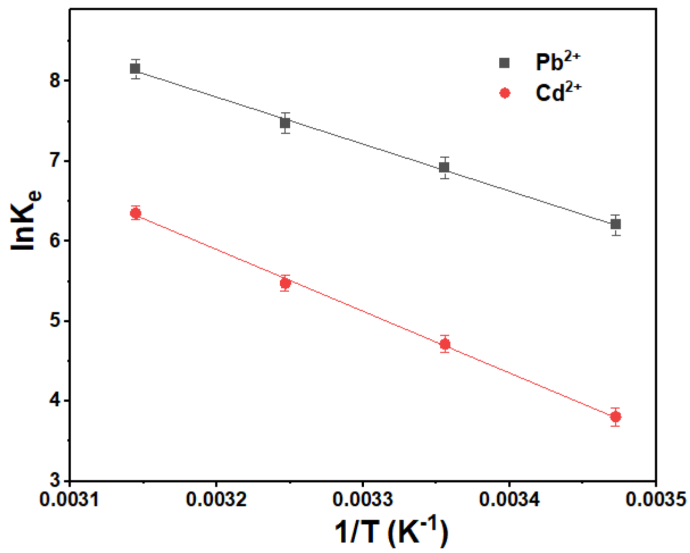

Figure S2: Plot of lnK_e versus 1/T for determination of reaction enthalpy (ΔH) and entropy (ΔS) of metal ions adsorption onto the CNTs-G5.

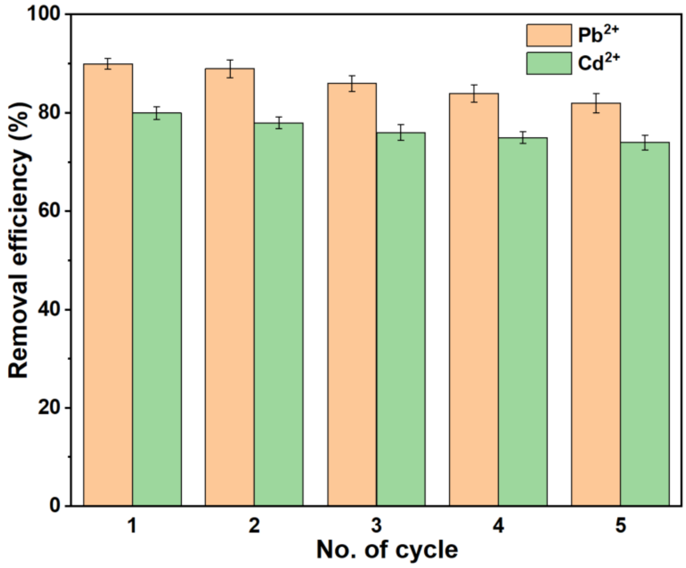

Figure S3: Recyclability results of CNTs-G5 for metal ion removal.

Table S1: Equilibrium rate constants, amounts adsorbed at equilibrium, and correlation coefficients for the adsorption of metal ions on CNTs-G5.

Metal ion	C (mg/L)	$q_{e,\,exp}$ (mg/g)	Pseudo-first order				Pseudo-second order			
			$q_{e,\,cal}$ (mg/g)	$k_1 \times 10^4$ (1/min)	ARE	R^2	$q_{e,\,cal}$ (mg/g)	$k_2 \times 10^4$ (g/mg·min)	ARE	R^2
Pb^{2+}	40	38.9±2.13	20.3	148.1	0.221	0.9785	39.0	301.1	0.073	0.9981
	60	41.9±2.24	19.4	147.9	0.311	0.9703	42.1	231.9	0.043	0.9926
	80	60.3±2.15	35.2	278.2	0.265	0.9615	61.4	151.4	0.094	0.9925
Cd^{2+}	40	34.3±2.34	19.8	190.1	0.145	0.9585	34.7	236.4	0.074	0.9960
	60	38.8±3.04	28.6	143.1	0.161	0.9740	37.9	139.9	0.091	0.9983
	80	55.8±3.67	41.6	145.9	0.194	0.9718	56.2	136.0	0.088	0.9973

Table S2: Adsorption isotherms constants for the adsorption of metal ions onto CNTs-G5.

Isotherm model	Equation	Parameter	Pb^{2+}	Cd^{2+}
Freundlich	$\ln q_e = \ln K_F + \frac{1}{n} lnC_e$	K_F (mg/g(L/mg)$^{1/n}$)	40.7158	32.5129
		$1/n$	0.1003	0.1142
		R^2	0.6985	0.7066
		ARE	0.346	0.354
Langmuir	$\frac{C_e}{q_e} = \frac{1}{K_L q_{max}} + \frac{C_e}{q_{max}}$	K_L (L/mg)	595.6731	0.8439
		q_{max} (mg/g)	80.7102	66.8003
		R^2	0.9984	0.9997
		ARE	0.067	0.056

Table S3: Comparison of various adsorbents for Pb^{2+} and Cd^{2+} adsorption.

Materials	Environmental aspect	q_{max} for Pb^{2+} (mg/g)	q_{max} for Cd^{2+} (mg/g)	Recyclability	Reference
oxidized multiwalled carbon nanotubes (O-MWCNTS	requires oxidants	5.73	3.34	N/A	[2]
CNT/starch/Fe_3O_4	physical deposition, use of metals	50.36	44.72	high (7 cycles)	[3]
waste biomass–based biochar and alginate-based biopolymer	physical, mixing, low stability	55	36.8	N/A	[4]
nitric acid-modified coconut shell carbon (MHBC)	high temperature	160.41	47.46	high (5 cycles)	[5]
Caragana korshinskii biochar (CB)	high temperature	220.94	42.43	N/A	[6]
cerium oxide modified activated carbon (Ce/ AC)	high temperature, use of metals	4.17	5.88	N/A	[7]
CNTs-G5	mild solvent, no oxidants and metals	80.7102	66.8003	high (5 cycles)	this study

Table S4: Determination of thermodynamic parameters for the adsorption of metal ions

Metal ions	*T* (K)	K_e (L/mol)	Δ*H* (kJ/mol)	Δ*S* (J/K·mol)	Δ*G* (kJ/mol)	R^2
Pb^{2+}	288	494.8757	48.894	221.345	−14.857	0.9981
	298	1006.4484			−16.557	
	308	1761.7712			−17.898	
	318	3474.6344			−19.524	
Cd^{2+}	288	44.9053	64.234	254.591	−9.110	0.9984
	298	111.5431			−11.289	
	308	238.4793			−13.109	
	318	574.4604			−15.214	

References

1. Valenzuela, F.; Quintana, G.; Briso, A.; Ide, V.; Basualto, C.; Gaete, J.; Montes, G. *J. Water Process Eng.* **2021,** *40,* 101951. doi:10.1016/j.jwpe.2021.101951
2. Li, X.; Cui, Y.; Du, W.; Cui, W.; Huo, L.; Liu, H. *Appl. Sci.* **2024,** *14,* 1745. doi:10.3390/app14051745
3. Zhang, M. *Biomass Convers. Biorefin.* **2024,** *15,* 8487–8500. doi:10.1007/s13399-024-05594-2
4. Biswas, S.; Sharma, S.; Siddiqi, H.; Meikap, B. C.; Sen, T. K.; Khiadani, M. *Water, Air, Soil Pollut.* **2021,** *232,* 8. doi:10.1007/s11270-020-04951-x
5. Qin, H.; Shao, X.; Shaghaleh, H.; Gao, W.; Alhaj, H. Y. *Agronomy (Basel, Switz.)* **2023,** *13,* 1813. doi:10.3390/agronomy13071813
6. Wang, T.; Zheng, J.; Liu, H.; Peng, Q.; Zhou, H.; Zhang, X. *Environ. Sci. Pollut. Res.* **2021,** *28,* 13800–13818. doi:10.1007/s11356-020-11571-9
7. Kouotou, D.; Ghalit, M.; Ndi, J. N.; Pastrana Martinez, L. M.; El Ouahabi, M.; Mbadcam Ketcha, J.; Gharibi, E. K. *Environ. Monit. Assess.* **2021,** *193,* 467. doi:10.1007/s10661-021-09267-9
